# Machine learning based personalized drug response prediction for lung cancer patients

**DOI:** 10.1038/s41598-022-23649-0

**Published:** 2022-11-07

**Authors:** Rizwan Qureshi, Syed Abdullah Basit, Jawwad A. Shamsi, Xinqi Fan, Mehmood Nawaz, Hong Yan, Tanvir Alam

**Affiliations:** 1 College of Science and Engineering, Hamad Bin Khalifa University, Doha, Qatar; 2 FAST National University of Computer and Emerging Sciences, Karachi, Pakistan; 3 Department of Electrical Engineering, City University of Hong Kong, Kowloon, Hong Kong; 4 Center for Intelligent Multidimensional Data Analysis (CIMDA), City University of Hong Kong, Kowloon, Hong Kong; 5 Department of Biomedical Engineering, The Chinese University of Hong Kong, Shatin, Hong Kong, SAR China

**Keywords:** Computer science, Lung cancer

## Abstract

Lung cancers with a mutated epidermal growth factor receptor (EGFR) are a major contributor to cancer fatalities globally. Targeted tyrosine kinase inhibitors (TKIs) have been developed against EGFR and show encouraging results for survival rate and quality of life. However, drug resistance may affect treatment plans and treatment efficacy may be lost after about a year. Predicting the response to EGFR-TKIs for EGFR-mutated lung cancer patients is a key research area. In this study, we propose a personalized drug response prediction model (PDRP), based on molecular dynamics simulations and machine learning, to predict the response of first generation FDA-approved small molecule EGFR-TKIs, Gefitinib/Erlotinib, in lung cancer patients. The patient’s mutation status is taken into consideration in molecular dynamics (MD) simulation. Each patient’s unique mutation status was modeled considering MD simulation to extract molecular-level geometric features. Moreover, additional clinical features were incorporated into machine learning model for drug response prediction. The complete feature set includes demographic and clinical information (DCI), geometrical properties of the drug-target binding site, and the binding free energy of the drug-target complex from the MD simulation. PDRP incorporates an XGBoost classifier, which achieves state-of-the-art performance with 97.5% accuracy, 93% recall, 96.5% precision, and 94% F1-score, for a 4-class drug response prediction task. We found that modeling the geometry of the binding pocket combined with binding free energy is a good predictor for drug response. However, we observed that clinical information had a little impact on the performance of the model. The proposed model could be tested on other types of cancers. We believe PDRP will support the planning of effective treatment regimes based on clinical-genomic information. The source code and related files are available on GitHub at:  https://github.com/rizwanqureshi123/PDRP/.

## Introduction

Lung cancer is a leading cause of deaths worldwide^1^, and has the lowest survival rate among all cancer types. It is the second most common type of cancer, and often diagnosed at later stages when metastatic spread to other parts of the body may have occurred^2,3^. In the last decade, rapid progress has been made in the management of non-small cell lung cancer (NSCLC) patients. Molecular targeting has made great advances, and epidermal growth factor receptor (EGFR) and ErbB family members have been identified as useful therapeutic targets^4^. Over-expression of EGFR is found in about 60% of advanced NSCLC patients^5^. The US Food and Drug Administration (FDA) has approved three generations of small molecule tyrosine kinase inhibitors (TKIs) Gefitinib/Erlotinib/Afatinib/Osimertinib as a first line treatment for lung cancer patients harboring EGFR mutations^6^. These TKIs produced encouraging results at the initial stage of therapy and increased the survival rate and quality of life of patients^7^. However, resistance to these drugs has appeared in many cases^8^. A major cause of this resistance is a secondary point mutation in the kinase domain of EGFR^9^.

Several studies have attempted to decode the mechanism of drug resistance in EGFR-mutated lung cancer^10,11^. These studies found many reasons, such as the secondary point mutation T790M^12^, breaking of the hydrogen bond at site 790, and reactivation of AKT^11^. *In silico* methods have been extensively applied to study these drug resistance mechanisms^3,13^. Molecular dynamics (MD) simulation^14^ is a computational tool, which has been used to understand dynamics^15^, stability^16^, and structural variations^17^. Recently, a framework has been developed for the visualization of protein-drug interactions in the analysis of drug resistance in lung cancer^18^. There is still much unexplained variation in patients’ responses to these drugs and clinical-genomic features of patients may play a significant role underlying the mechanism of drug resistance^19^ and patient stratification^20^.

**Figure 1:**
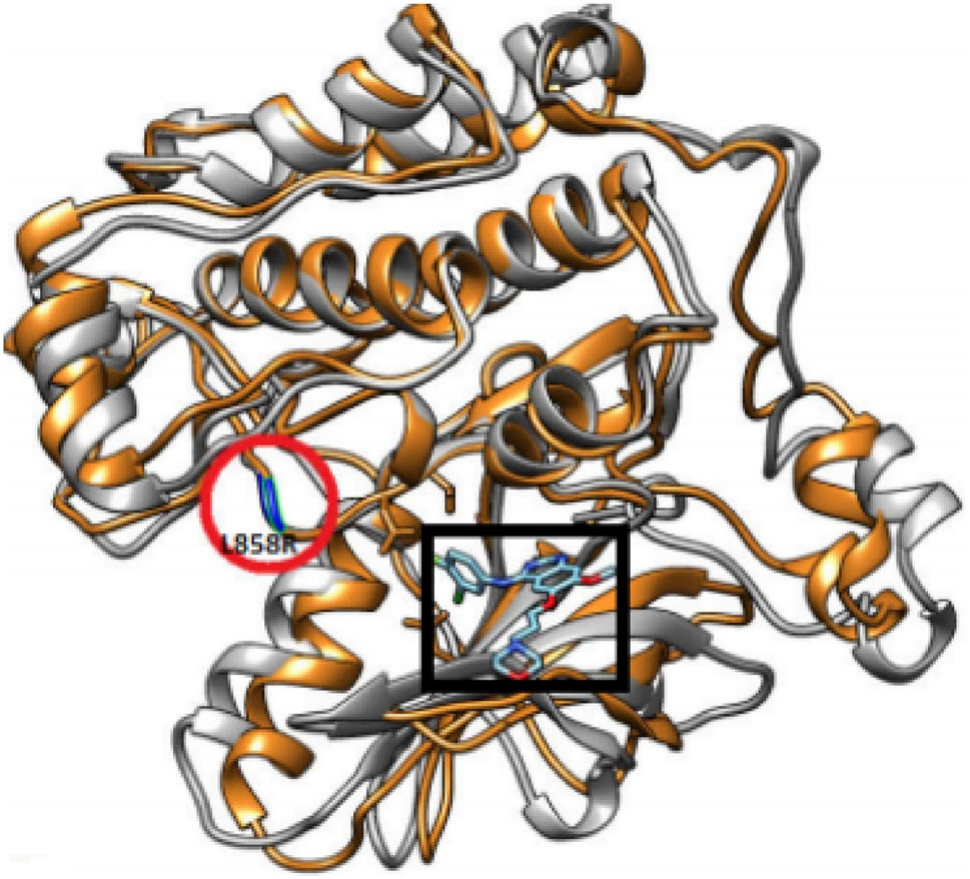
An EGFR-Gefitinib complex modeled with the L858R mutation. The drug molecule is indicated by the black square and the mutation by the red circle. The image was generated using PyMol.

The completion of the human genome project^21^ allowed a shift of paradigm from the traditional medical model of targeting large populations to precision therapies^22^. Information from genomics and electronic health records provides novel opportunities for patient care, prevention, and devising optimal treatment strategies^23^. Predicting a patient’s response to a drug treatment, or identifying their optimal treatment strategy, such as the combinations and doses of drugs, is challenging for computational methods due to limited data sources, disparity among labels and unknown biological evidence^24,25^. The position of the drug-binding site, binding free energy, geometrical features, and clinical information may be used to model multi-class drug responses^13,26,27^. The protein data bank (PDB)^28^ contains several high-resolution structures of EGFR bound to different generations of approved drugs, providing opportunities to build structure-based data-driven models. An EGFR-L858R dimer with Gefitinib is shown in Fig. 1.

As drug responses are often mediated by protein-drug interactions, the geometry of the drug-target binding site or pocket can be a useful predictor of drug response. MD simulation of the binding energy of drug-mutant complexes and related personal characteristics of patients, when used as input to an extreme learning machine (ELM)^29^, classified drug response levels into two classes^13^, with an accuracy of 95.3%. Combining local geometrical properties with energy related features in an Eigen binding site method achieved an average accuracy of 69.35% in predicting four classes of drug responses^26^. Patients’ demographic and lifestyle patterns and geometric features of drugs were used for a similar predictive model^30^, and protein-drug interaction footprint tensors were used in a three-level drug response predictive model^31^.

While these studies demonstrate the potential of combining dynamic molecular features with patient data to predict responses to a drug, the quality of the predictions needs to be improved if they are to be of clinical use. In this work, we combined the geometric information of the drug-target binding site, the binding energy, and patient’s clinical information to predict four classes of drug response with an imbalanced dataset. The proposed personalized drug response prediction (PDRP) model achieves state of the art performance in predicting drug responses. The main contributions of this work are:

- We have developed a drug response prediction model using molecular dynamics (MD) simulation and machine learning (ML), which achieves state of the art performance.
- We have proposed a set of novel geometric features by modelling the binding site of drug-target complex. Our work demonstrates the contribution of the geometrical features of binding sites in improving the predictive accuracy of our ML model.
- The proposed model, PDRP, is a generic concept, which can be used for other cancers or diseases with minor modifications, with potential applications in clinical decision support systems and patient stratification.

## Results

In this work, we have developed a drug-response predictive model for lung cancer patients. Demographic and clinical information, such as age, gender, survival time, smoking history, tumor progression level and type of mutation, were collated from previous studies^13,26,32–34^. We simulated EGFR-Gefitinib complexes in the AMBER software suite^35^ for 2 ns and extracted the trajectories. Based on the simulated trajectories, we proposed novel geometrical features $x_{g1}$ (the matching rate, number of connected atoms, and number of hydrogen bonds) and $x_{g2}$ (the number of convex atoms and Euclidean distance) from the EGFR mutant-drug complexes to predict the drug-response level as one of complete response, partial response, stable disease or progressive disease. We also used binding free energy as a feature in the machine learning model. The combination of clinical, geometric and energy related features boosted the performance of the machine learning model.

### Baseline statistics

201 NSCLC patients form the cohort of this study. The patients had a median age of 63 years, 35% (71) were female and 65% (130) male, and about 75% were non-smokers. All patients received EGFR-TKIs as their first line of treatment. Response levels 0 and 1 indicate complete and partial responses to the drug. Response levels 2 and 3 correspond to stable and progressive disease (No response). The dataset used here consisted of 19, 118, 30, 34 patients at response levels 0, 1, 2 and 3, corresponding to complete, partial, stable, and progressive disease (No response), respectively.

### Proposed feature set summary

**Figure 2:**
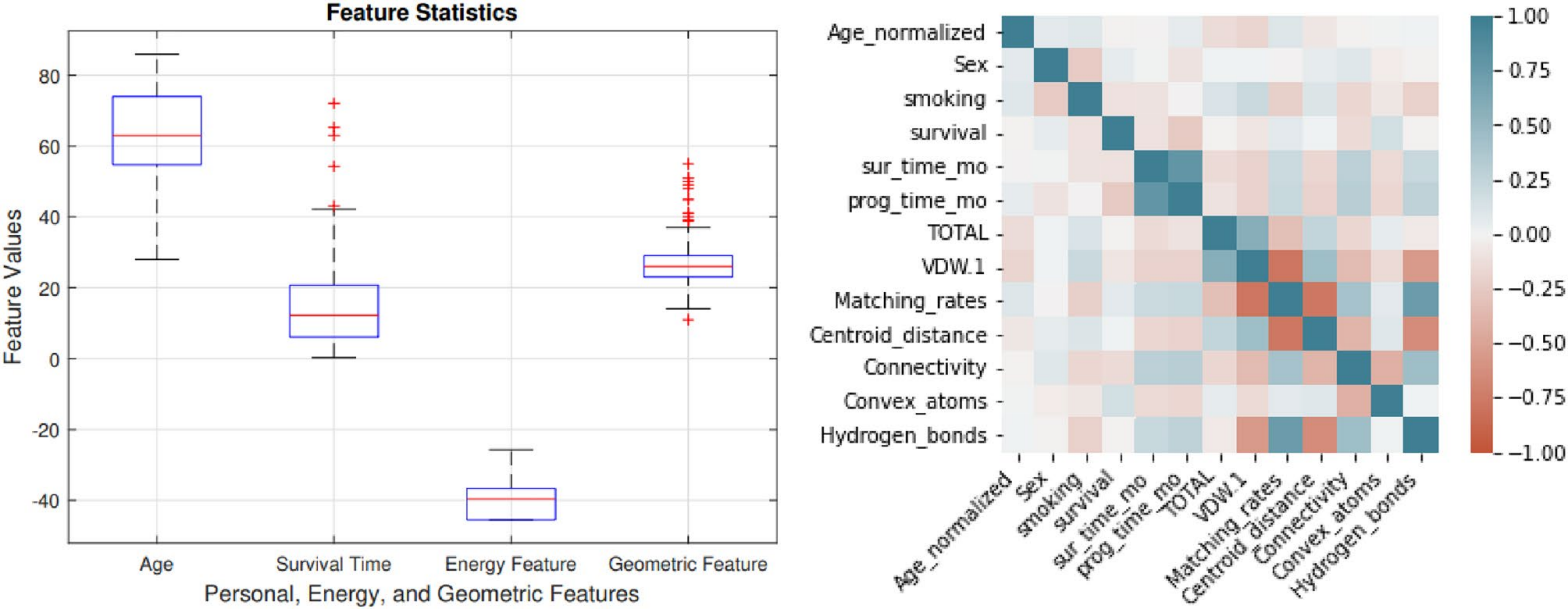
Box plot of normalized values for energy, and geometrical features (left panel), and correlation among features (right panel).

For a patient, his/her demographic and clinical information (DCI), the energy and geometric features of their mutant EGFR-Gefitinib complex were obtained and used to predict their drug response level through machine learning classifiers. A detailed description of the features and their value ranges are presented in Table 1. In total we extracted 4, 4, and 5 features from DCI, energy and geometric types, respectively. Figure 2 shows the box plot for different features and the correlations among them.

**Table 1:** Clinical information, energy and geometrical features: description and values.

Feature type	Attributes	Description	Discrete/Continuous	Range
DCI	Age	Patient’s personal information	Discrete	[0–4]
	Sex		Discrete	[0–2]
	Smoking history		Discrete	[0–2]
	Response status		Discrete	[0–3]
Energy	VDW	Van der Walls energy	Continuous	[− 60 to − 45]
	EEL	Electrostatic interactions	Continuous	[− 23–11]
	ESURF	No polar component of the solvation energy	Continuous	[− 45 to − 1]
	EPB	Polar component of solvation free energy	Continuous	[27–40]
Geometric	Matching rates	Matched atoms	Discrete	[0, 17]
	Convex atoms	Strength of interaction	Discrete	[0, 43]
	Connectivity	Connected atoms	Discrete	[0, 23]
	Euclidean distance	Distance between drug and target	Continuous	[30–39]
	Hydrogen bonds	Number of hydrogen bonds	Discrete	[775–1650]

**Figure 3:**
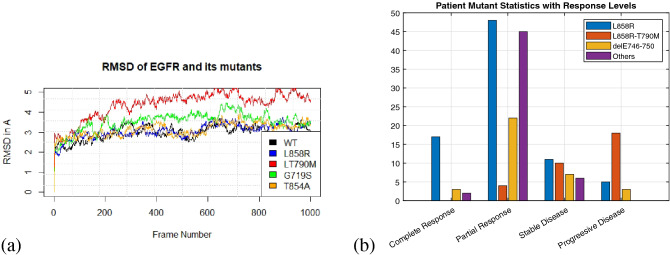
(**a**) MD trajectories of EGFR and some mutants showing RMSD from the reference structure. As the values are below 5, the structures are reliable for further analysis. (**b**) Distribution of disease response classifications for 201 patients by the three most common mutations (L858R, L858R-T790M, del E746-750), and the others.

**Figure 4:**
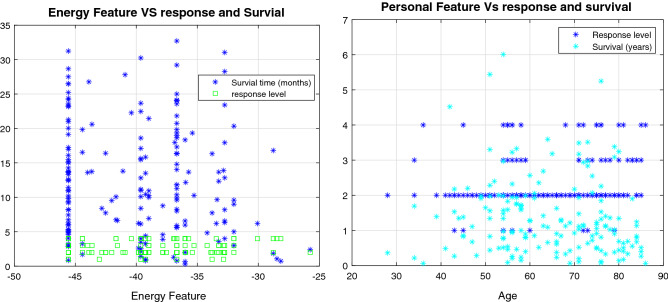
Disease response classification and survival time (months) by binding free energy (left panel) and disease response classification and survival time (years) by age of patient (right panel).

A total of 33 different EGFR mutations occurred in these patients. The root mean square deviations (RMSD) of the trajectories from MD simulations of WT and four mutants and the patient disease response classification by mutation type are shown in Fig. 3. The most common mutations were L858R, delE746−750 and L858R−T790M. All mutations were modelled based on the EGFR 3D structure using Rosetta^36^. The potency of an inhibitor can be measured by the time a patient survives and their drug response level. The relationship between the drug response and the personal and energy features were not linearly, or one-to-one, related to drug response or survival time (Fig. 4). Drug response was classified into four levels, based on the response evaluation criteria in solid tumors (RECIST)^37^. A list of mutations types is provided in the Supplementary File S1.

### Performance of the proposed PDRP model

**Figure 5:**
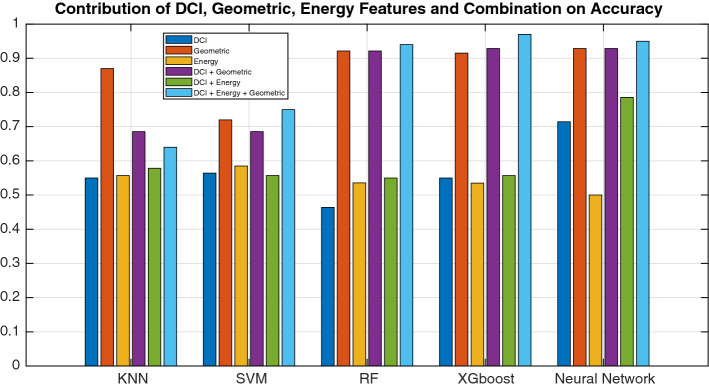
Contribution of geometrical, DCI, and energy-related features to the accuracy of the model.

To build the PDRP model, we incorporated clinical information, MD simulation results and novel geometric features from the protein–drug interactions. An ablation study, based on each type of feature and the corresponding model’s performance, showed that the geometrical features were the most powerful predictors, followed by DCI and energy-related features (Fig. 5). Of the models tested, XGboost had the best performance. Demographic and clinical features brought a little improvement to the performance, and the combination of all three types of features boosted the performance in XGboost, Random Forest and the Neural Network.

**Figure 6:**
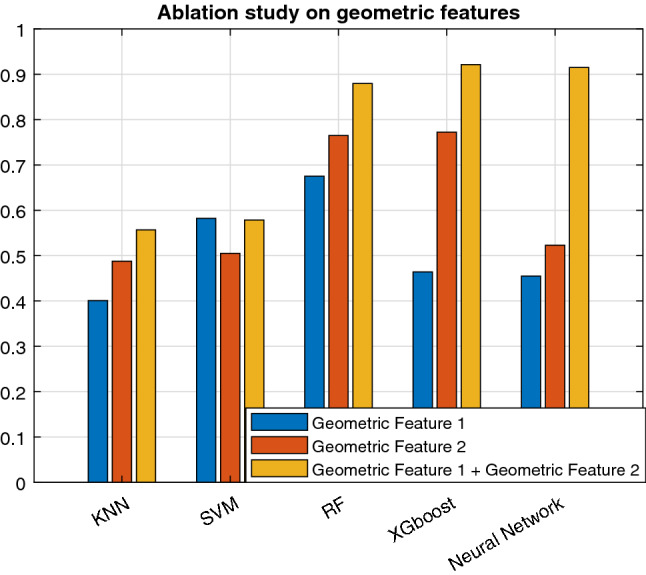
Ablation study on geometric features.

**Figure 7:**
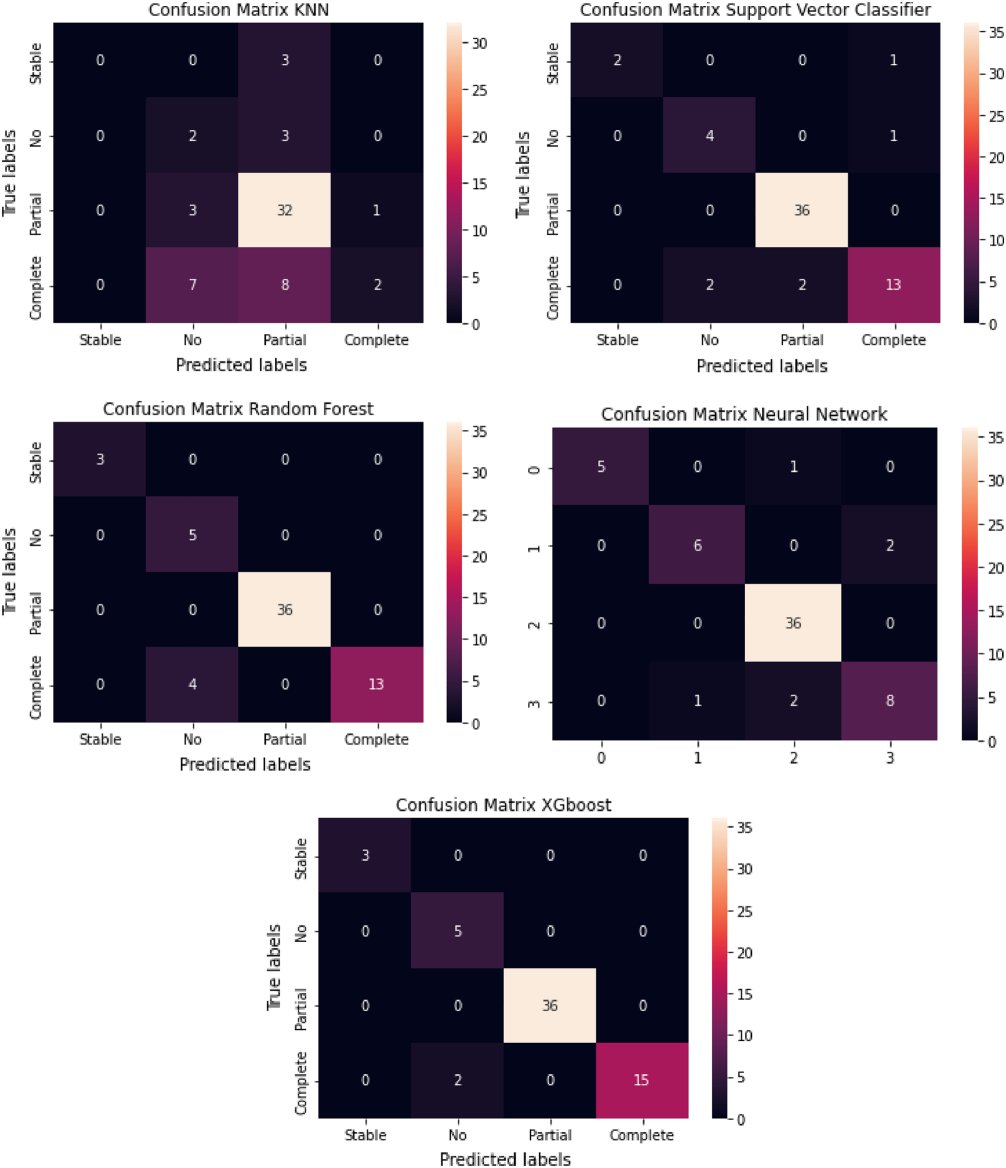
Confusion matrix for testing dataset.

**Figure 8:**
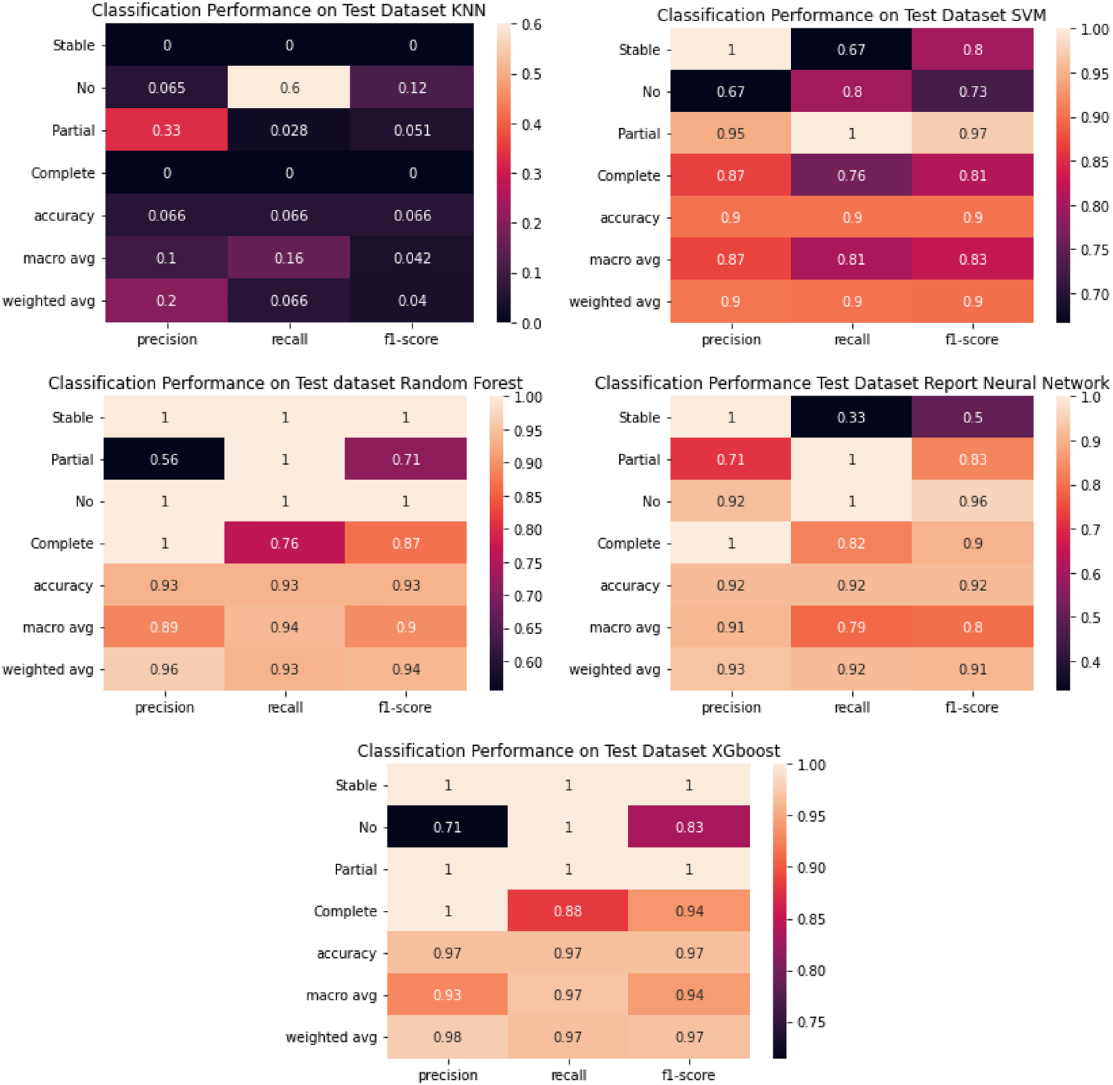
Classification performance on testing dataset.

The confusion matrices for the training and classification reports are shown in Figs. 7 and 8 , respectively. The XGboost classifier achieved 97.5% accuracy, 97% recall, 93% precision, and 97% F1-score with only two mis-classifications for an independent testing set of 61 samples. The performance of the random forest and neural network and were close to, but not better than, XGboost. The classifiers were trained using a nested-cross validation approach, and the parameters of classifiers were optimized using a grid search approach.

## Discussion

Our main focus in this work was on modeling the geometry of the drug-binding pocket and combining this with the demographic and clinical information (DCI) of the patients. The geometrical features (the number of convex atoms at the interaction surface of the complex, the matching rates of surface atoms, the dynamic distances between the center of the drug molecule and binding-site residues throughout the trajectory, and the number of hydrogen bonds) were the most discriminative features. Combining clinical and molecular predictors to identify drug-sensitive patients was most effective. The mutation changes the shape and structure of the drug binding complex, which leads to changes in the values of the geometrical features and thus the drug response. Further investigation of this model may result in geometrical features that better stratify patients based on mutation, age or gender specific therapies and the model might also help in selecting the optimal drug for specific patients. In addition, we perform another ablation study only on geometric features. We found that geometric feature 2 has more predictive capability than geometric feature 1. However combining the feature further boosts the performance, as shown in Fig. 6. Moreover, It will be interesting to see the performance of modern geometrical based deep learning algorithms^38^, if a large number of samples are available.

### Comparison against other methods

Table 3 shows that the classification accuracy of the proposed method achieved state of the art performance relative to related works and achieved this while predicting the highest number of classes (four) of drug responses. Methods predicting fewer response levels performed better than earlier four-level predictors, however, our method outperformed all previous work. The combination of geometrical, energy, and personal features seems to be a more optimal strategy for predicting the drug response. The highly accurate pioneering work^13^ used binding free energy, and personal features of 168 patients but only predicted a two-class drug response. Figure 5 shows the contribution of each feature. The demographic and clinical (DCI) feature slightly improved the drug response prediction as shown in Table 2. To further test the performance of the proposed model, we convert our problem into 2 class binary classification. We combined partial response and full response to one class, and stable disease and no response to another class. After that, we re-run the method proposed by^13^ and^39^ on our task. The results are shown in Table  3. Our method also performs well on binary classification problem.

**Table 2:** Comparison of PDRP with other methods.

Reference	Patients	Features	Method	Response level	Accuracy
^13^	167	Personal and energy	Extreme learning machines	2	95.13
^39^	355	Personal and genetic	Sequential minimization optimization	2	76.56
^30^	137	Geometrical and personal	Softmax regression	4	70.78
^26^	311	Energy and geometrical	Support vector machine	4	69.35
^31^	NA	Protein-drug interactions	Naive Bayes	3	95.50
PDRP	201	DCI, Energy, Geometric	XGboost	4	**97.50**

The best accuracy is in bold.

The 2-class response includes complete response and no-response, while 3-class response includes low, moderate, and full response. The 4-class response includes complete-response, partial-response, no-response, and progresive disease.

**Table 3:** Comparative results on binary class problem.

Reference	Method	Accuracy
^13^	Extreme learning machines	89.13
^39^	Sequential minimization optimization	79.58
PDRP	XGboost	**98.50**

The best accuracy is in bold.

We converted partial response and full response to a single class “response”; and no response and stable disease to a single class “no response”.

### Performance of PDRP on gender stratified samples

**Figure 9:**
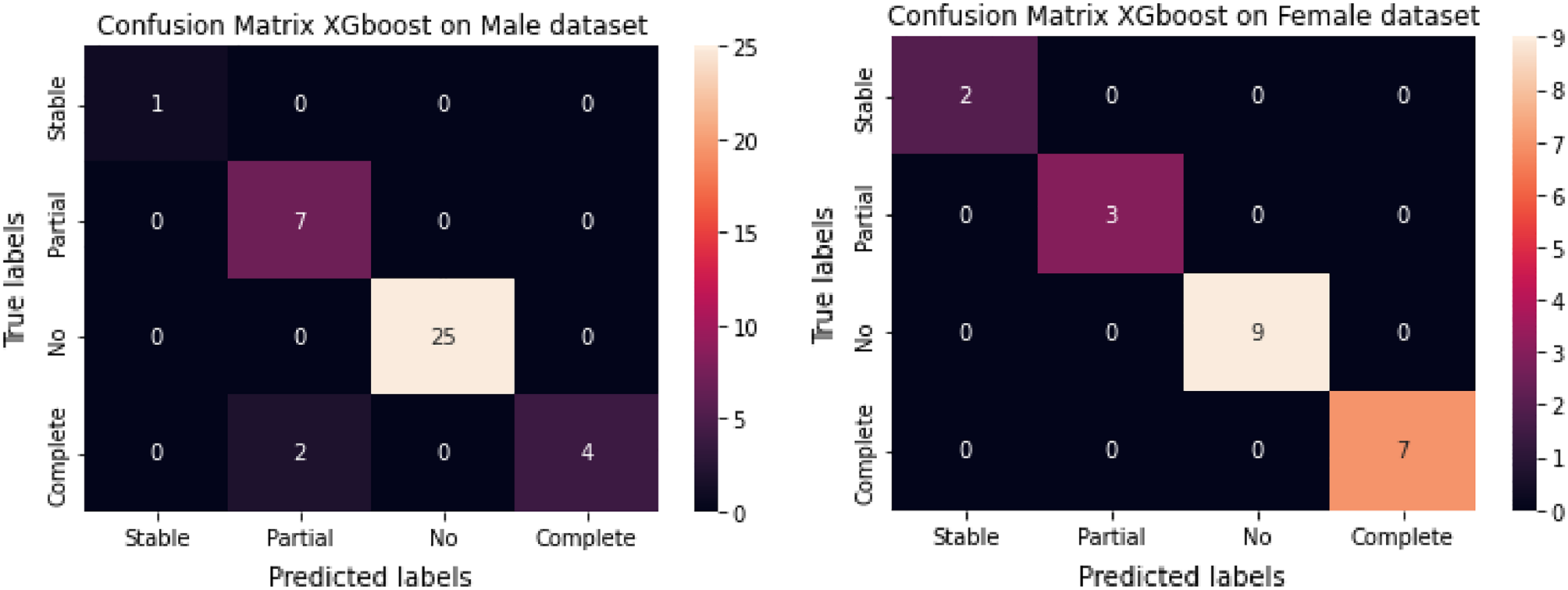
Confusion matrices for the XGBoost model on gender-stratified patients.

Experiments using PDRP on gender stratified samples showed that the model performed well on both male and female patients (Fig. 9). Drug responses were correctly identified in 21 females patients with no mis-classification. However, there were two mis-classifications, between partial and complete responses, in the male dataset of 39 patients.

### Translation research

In this paper, we proposed two novel composite features. Composite feature $x_{g1}$ captures the interaction strength of the protein-drug complex, and composite feature $x_{g2}$ captures the shape and local surface information of the protein-drug complex. The morphological properties of the binding site, the number of convex atoms and the dynamic distance help us to model the geometrical properties. Length and strength of protein-drug interactions are represented by these features, which helps in predicting the drug-response. The features that play a prominent role in the drug response could be further studied to screen inhibitors for personalized therapy and may become useful biomarkers for drug screening. We also showed that combining DCI, geometrical and energy related features helps to boost the performance of the drug response prediction model.

### Limitations

A limitation of this study is that it contained only the 33 most common EGFR mutations from the 594 EGFR mutations available in COSMIC database^40^. However, the mutations studied account for about 90% of all mutations. It is difficult to determine drug sensitivity to rare mutations due to limited patient data. Another limitation is the small dataset of 201 patients. Obtaining clinical data is difficult due to privacy and ethical considerations. Most clinical studies consist of fewer than 400 patients (Table 3) and may have imbalanced numbers of patients at each response level. Despite this, our model achieved a highly accurate prediction rate.

Protein-ligand interactions usually take place in nanoseconds, so our simulation is adequate. Many of the previous studies on EGFR drug response prediction also used 2-ns MD simulation^13,31^. However, the time scale is highly case-specific and may cause computing costs to explode. Of course, it would be useful to study the interactions for longer time, but MD simulations only provide an approximate solution, and the later part of the simulations can be less accurate. Quantum mechanics (QM)^41^ based analysis would be more accurate, but the computational cost is currently beyond the reach of most computers unless we only deal with very small molecules.

## Methods

**Figure 10:**
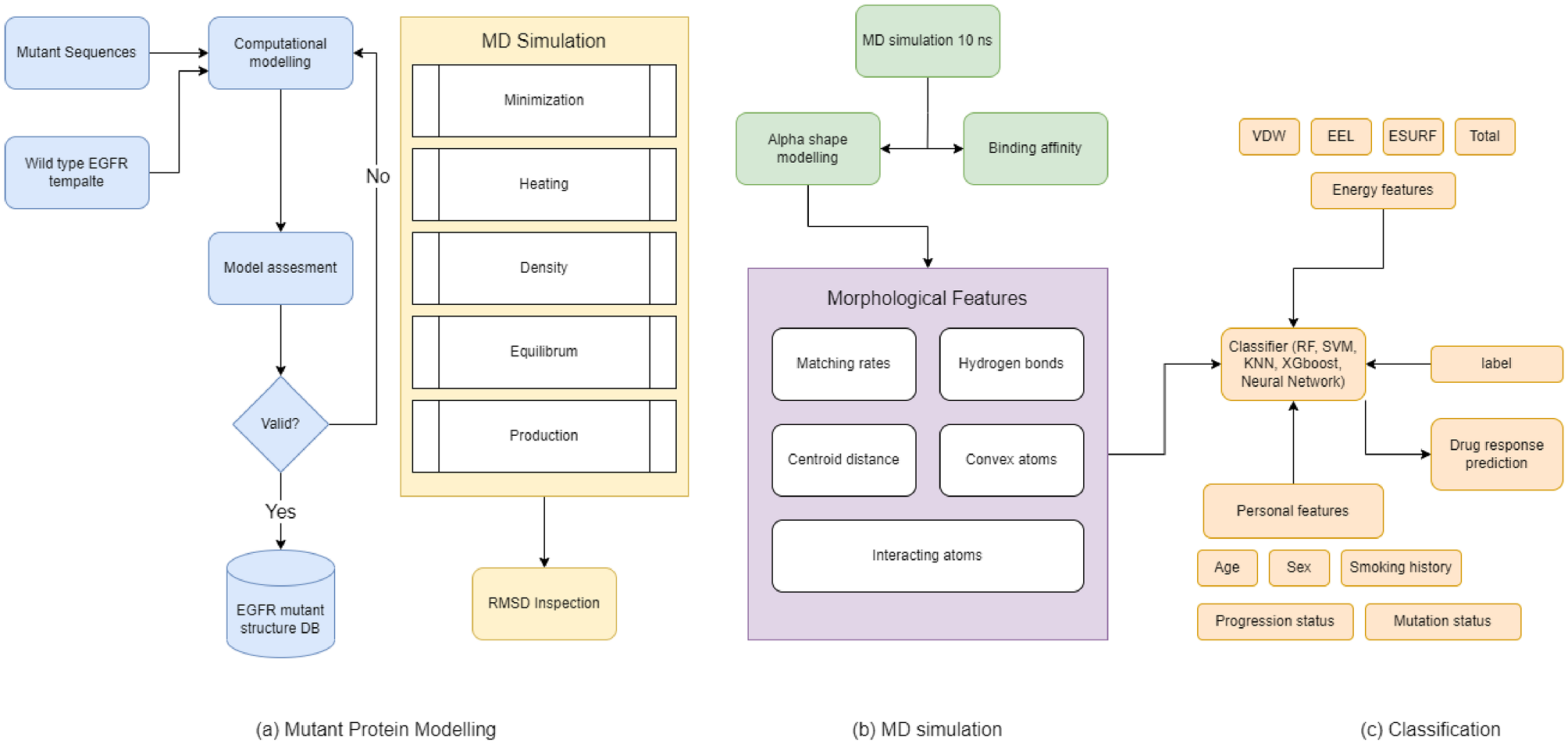
The framework for predicting the drug response in lung cancer patients based on personal data, binding energy, and geometric features. Mutant structures are predicted by computational methods then molecular dynamics simulations extract energy and geometrical features. Machine learning classifiers then predict four classes of drug response from these features.

The framework to classify individual patient outcomes is divided into three modules: computational modeling of mutant/drug structures, MD simulations, and classification. Figure 10a shows a method for computationally modeling the structure of the EGFR-Gefitinib drug complex; PDB 2ITY was used as the template structure. Figure 10b and c show the steps of the MD simulation and the formulation of the classification models. Each mutation was modeled in the 3D structure of the protein-drug complex. Our dataset consisted of 33 different EGFR point mutations. For each mutant, MD simulations were performed and the binding free energy was calculated. Multiple machine learning models were used to predict the response level.

### Dataset collection

All the methods under this study were performed in accordance with the relevant guidelines and regulations of City University of Hong Kong (CityU). Informed consent was taken from all participants.The experimental protocols were approved by the Institutional Review Board (IRB) of City University of Hong Kong. The clinical information used in this study was collected from several published sources^13,26,32–34^. A dataset of 201 NSCLC patients was obtained and 140 samples were used to train the model. 61 independent samples were used to test the model, with 3, 5, 36, and 17 samples for stable disease, no response, partial response and complete response respectively. The L858R mutation was the most frequent mutation, followed by secondary point mutation L858R-T790M and delE746-750, as shown in Fig. 3b. The EGFR mutations were selected based on a survey in^42^.

### Demographic and clinical information (DCI)

Demographic information such as age, sex, smoking history as well as clinical information such as survival [0,1], drug response level and performance status were extracted from the clinical dataset. Age was encoded between 0 to 4 based on the age brackets (0,40), (41, 50), (51, 60), (61, 70), and (71 and above), due to inherent modes.

### Modeling of 3D structures

The 3D mutant structures were predicted based on the crystal structure of wildtype EGFR taken from Protein Data Bank (PDB)^28^ with PDB ID 2ITY. The high resolution ddgmonomer (HRDM)^43^ protocol in Rosetta was used to predict point mutations, and the comparative modeling protocol was used to predict multi-point mutations^44^. Quality assessment of predicted structures was performed by Verify3D^45^, and Q-mean^46^.

### Molecular dynamics simulation

MD simulations of the protein-drug complex were performed using the QM/MM method in Amber^47^ with a surrounding waterbox neutralized using Na+ and Cl- atoms and the ff9SB^48^ and GAFF force fields. The total energy of the system was the summation of bonded (stretch, bend, torsion) and non-bonded (electrostatic, van der Waals) terms.

1 $$\begin{aligned} E_{total} = E_{stretch} + E_{bend} + E_{torsion} + E_{electrostatic} + E_{vdw} \end{aligned}$$

Energy minimization was used to refine the modeled structure before the MD run, which begins with the system being heated from 0K to 300K, followed by equilibration at constant pressure and density for 500-ps and 50-ps respectively. The SHAKE algorithm^49^ was utilized to control temperature and constrain bond stretching. After reaching a stable state, MD simulation runs were carried out for 2-ns at a steady temperature (300K) and pressure (1 Atm). A twelve core 3.47 GHz CPU with eight GB RAM was used to run MD simulations.^50^, with a Tesla C2075 GPU^51^ used in production runs. Each simulation took roughly 12 h to complete. The trajectory was extracted using the CPPTRAJ^52^ package in Amber, with frames gathered every 10-ps, producing 200 frames per run.

#### Binding free energy

The free energy of binding^53^ of a drug to a protein in a solvated environment estimates the binding affinity^54^. The parallel version of MM-GBSA^55^ on a twelve core, 3.47 GHz CPU was used for the simulation. The MD trajectory was considered as input to MM-GBSA. Each round of simulation took on average twelve hours for computation. The binding free energy is calculated based on the theory of the thermodynamic cycle in vacuum and solvated environments^56^ as:

2 $$\begin{aligned} \begin{aligned} \Delta {G} = \Delta {G}_{Bind, Vacuum} + \Delta {G}_{Solv, Complex} \\ - (\Delta {G}_{Solv, ligand} + \Delta {G}_{Solv, Receptor}) \end{aligned} \end{aligned}$$

where $\Delta {G}$ is the binding free energy difference of the receptor-ligand system in a vacuum. $\Delta {G}_{Solv, Complex}$, $\Delta {G}_{Solv, ligand}$, and $\Delta {G}_{Solv, Receptor}$ represent their energy differences between vacuum and solvent states. The energy component is composed of Van der Waals forces (VDW), electrostatic energy (EEL), the electrostatic contribution to solvation, and non-polar contributions to the solvation free energy (ESURF).

### Geometric features

Interactions between the binding site residues of a protein and small molecule inhibitors are commonly used in prediction methods^57^. Local geometric surface properties were determined based on the alpha shape^58^ using the computational geometry algorithm library (CGAL)^59^.

#### Convex atoms

Each atom in a drug-mutant system has a position and mass, represented as a = (*p, w*), where *p* is the position and *w* is the mass of the atom. Two atoms $\hbox {a}_1$ = (${p}_1$, ${w}_1$) and $\hbox {a}_2$ = (${p}_2$, ${w}_2$) are defined as orthogonal or sub-orthogonal using the following equation.

$$\begin{aligned} {\left\{ \begin{array}{ll} |p_{1}p_{2}| = w_{1} + w_{2}, &{} a_{1} \perp a_{2} \\ |p_{1}p_{2}| = w_{1} > w_{2}, &{} a_{1} \perp _{s} a_{2} \\ \end{array}\right. } \end{aligned}$$

From the alpha shape, the solid angle^60^ of atoms was determined to characterize the geometric properties of the local surface. If A, B, C, and D are the vertices of a tetrahedron, the solid angle, ${\Omega }_i$, is:

3 $$\begin{aligned} \Omega _i = \sum _i (\phi _i^{AB} + \phi _i^{BC} + \phi _i^{AC} - \pi ), \end{aligned}$$

where $\phi _i^{AB}$, $\phi _i^{BC}$, and $\phi _i^{BC}$ represent the dihedral angles of tetrahedron *i*.

4 $$\begin{aligned} \Omega ' = \frac{cos(\Omega _i)}{4}, \end{aligned}$$

**Figure 11:**
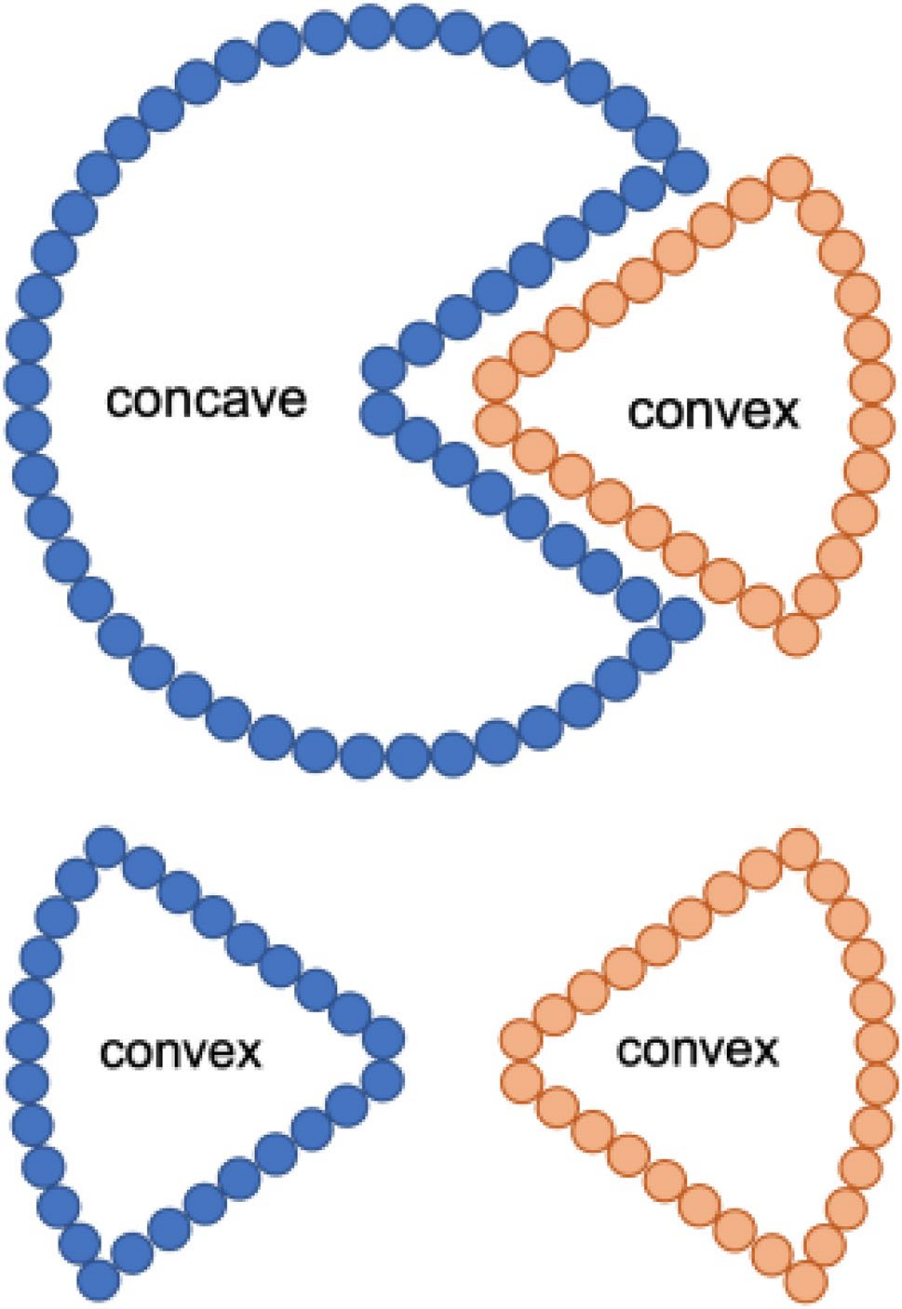
Atoms in convex and concave shapes at the surface curvature. The figure at the top shows a matched concave-convex pair, thus a strong interaction, while the figure at the bottom shows an unmatched pair and a weak interaction.

$\Omega '$ results in a convex shape if positive and a concave shape if negative (Fig. 11). The number of atoms in a convex shape at the local surface of the drug-dimer complex was used.

#### Matching rates

The atoms at the interface of the structures create the interaction between the drug and the target. The surface atoms were collected using the alpha shape algorithm, and named as point set A. After that, point sets B and C were obtained, to represent the surface atoms of the target and the drug, respectively. The interacting atoms (*I*) were obtained using set operations and further classified as, interacting atoms in the drug, $I_d$, or target, $I_t$, according to the following equation:

5 $$\begin{aligned} {\left\{ \begin{array}{ll} I = (B~\cup ~C) - A \\ I_t = (I~\cap ~B) \\ I_d = (I~\cap ~C).\\ \end{array}\right. } \end{aligned}$$

The matching rate was determined by selecting atoms at the drug and the target. If one of the atoms is convex and other is concave, the pair is recorded as matched and there is a strong interaction between them. If both atoms are convex or concave, the pair is unmatched, and the interaction is weak, as shown in Fig. 11. The matched and unmatched atoms are determined as:

6 $$\begin{aligned} f(B, C) = {\left\{ \begin{array}{ll} 1&{}~\Omega _B~\times ~\Omega _C~< 0 \\ 0&{}otherwise \\ \end{array}\right. } \end{aligned}$$

Matching rates are calculated for each frame of the MD trajectory as:

7 $$\begin{aligned} MR = \frac{\sum _{i, j}f(B_i, C_j)}{N} \end{aligned}$$

*MR* represents the matching rate, $f(B_i, C_j)$ is a matched atom pair, and *N* is the total number of MD snapshots. The matching rate is used as a feature in this work, and low matching rates were linked to low drug responses.

#### Connectivity measure

Connectivity changes between binding site residues and the drug molecule throughout the MD simulation. We define a local threshold value of 40 based on Euclidean distance and then record the number of atoms that remain within this threshold throughout the MD Simulation. The consistency of these connections may identify critical atoms and could be used as a predictor of the drug response level:

8 $$\begin{aligned} C_{k, i} = \sum _j A_{k, i , j} \end{aligned}$$

where $\hbox {A}_{k, i, j}$ represents the connection between the $i^{th}$ EGFR atom and the $j^{th}$ drug atom in the $k^{th}$ MD snapshot, and is 1 if there is a connection and zero otherwise. Let

9 $$\begin{aligned} D_{k} = \sum _i C_{k, i} > 0 \end{aligned}$$

which represents number of connected atoms in the MD snapshot. The number of connected atoms over the entire trajectory was used as a feature.

10 $$\begin{aligned} E_k = \frac{\sum {_{i = 1}^{N}} (D_{k, i})}{N} \end{aligned}$$

#### Binding site positioning

The positioning is evaluated using the Euclidean distance between the EGFR binding site atoms and the center of the drug-molecule.

11 $$\begin{aligned} D(a,b) = \sqrt{(x_a - x_b)^2 + (y_a - y_b)^2 +(z_a - z_b)^2} \end{aligned}$$

The binding site residues are represented by their alpha-carbon (CA) atoms. For example, if there are 14 CA atoms at the binding site, and two atoms at the drug molecule center, then a 14 $\times$ 2 or 28$~\times$ 1 vector will represent this. The distance over the entire MD simulation of 200 frames can be represented as a 200$~\times ~$28 matrix. The binding site position is represented as the average distance between the drug and the target:

12 $$\begin{aligned} D_{avg} = \frac{\sum _{i = 1}^N (D_i)}{N} \end{aligned}$$

where $\hbox {D}_{{avg}}$ shows the binding site position, $\hbox {D}_i$ shows the ith MD snapshot distance, and *N* is the number of MD snapshots. All the feature values were normalized to [0,1]. Generally, drug sensitive mutants have less distance between the drug and the target.

**Figure 12:**
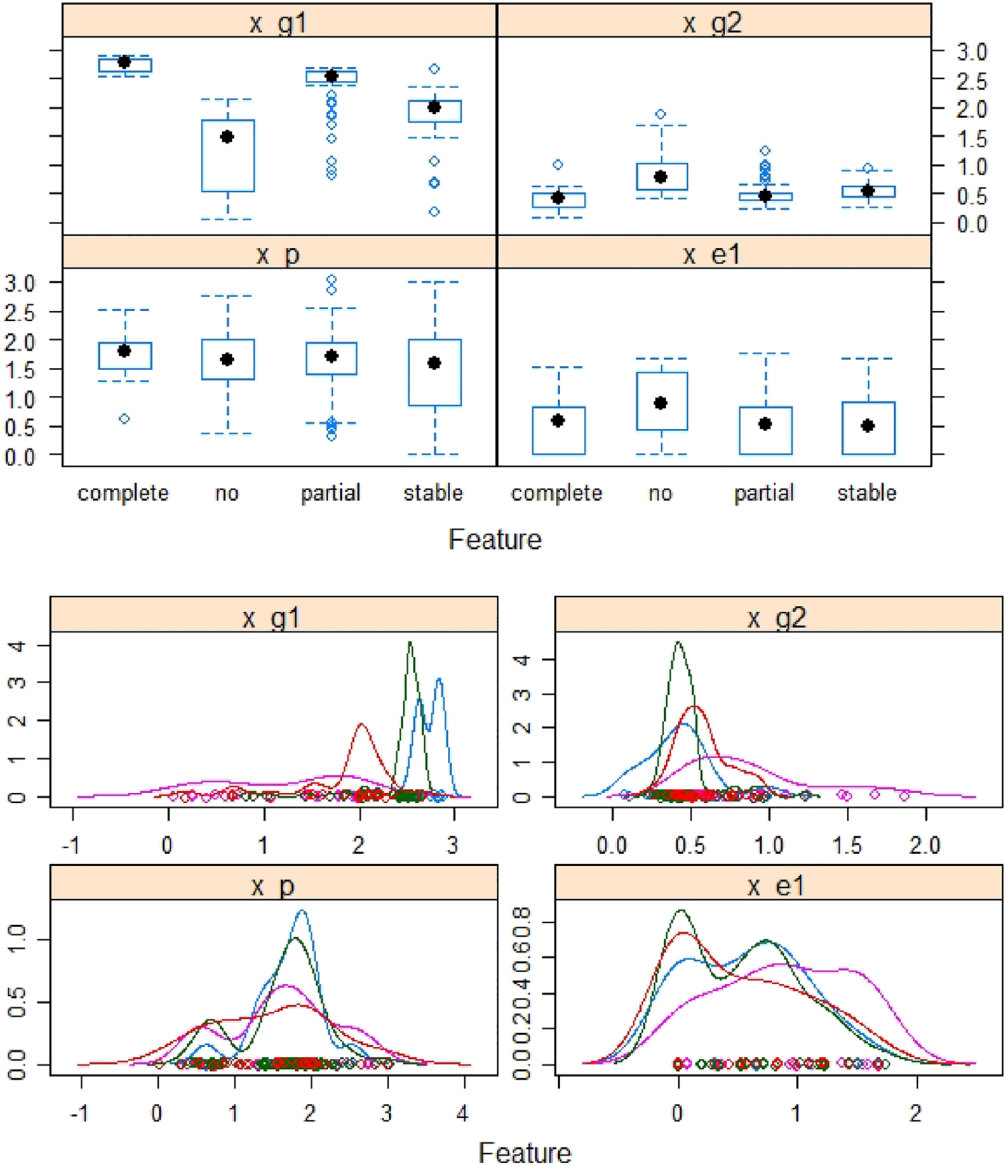
Geometric, energy and personal features and their distributions.

#### Hydrogen bonds

Hydrogen bonds contribute to the stability of a structure and can provide insights about interactions within the structure. Stable systems tend to have more hydrogen bonds. The number of hydrogen bonds in the EGFR-drug complex were calculated using the hbond command in Amber.

#### Composite geometric features

The geometric features were combined to make two composite features, with the matching rate, number of connected atoms, and number of hydrogen bonds as one feature, $x_{g1}$ and the number of convex atoms and Euclidean distance, $x_{g2}$, as the other. Similarly, the personal features and energy features were combined as $x_p$ and $x_{e1}$, respectively.

### Feature normalization

Each feature was normalized to the range [− 1, 1] using z-score normalization^61^.

13 $$\begin{aligned} z_i = \frac{x_i - \mu }{\sigma } \end{aligned}$$

where $z_i$ represents the normalized value, $\mu$ represents the mean value, and $\sigma$ represents the standard deviation value for each feature. Composite geometric features, energy features, clinical features and their density distributions are presented in Fig. 12.

### Development of the classification model

For patients with clinical information, geometrical and energy features were obtained from their EGFR mutant drug complex, and classifiers were trained to predict one of the four-classes of drug response level. Five popular classifiers i.e., KNN, SVM, Artificial Neural Network, Random Forest, and XGboost were tested using Python Scikit-learn^62^ and Tensorflow library^63^. We also used CARET package^64^ of RStudio for feature visualization plots. We used 141 samples for training the model. The parameters were optimized using a GridSearch approach. To ensure that the predictions made by the model are not by chance, we also performed y-scrambling and randomly shuffled the labels for 50 iterations. The mean accuracy drops down to 0.48587 from 0.975. To further avoid over-fitting and careful selection of hyper-parameters and model, we used a nested cross-validation function^65^, with an outer loop of 10 and inner loop of 3. The parameters are selected with a grid search of (10, 100, 500) estimators, (2, 4, 6, 10, 12) features and maximum depth of 3 for XGboost and Random Forest. The final proposed XGboost has a mean accuracy of 0.971 with a standard deviation of 0.035. The results vary between 0.8571 and 1 during cross validation. We also trained a neural network with four layers, with sigmoid activation for hidden layers and softmax for the output layer, for 8000 epochs. We used different activation functions, including Relu and dropout with different thresholds during the model selection. The categorical cross-entropy as the loss function, and RMSProp as an optimizer. We also implemented early stopping to monitor the validation loss with a patience value of 100. The source code is available on GitHub https://github.com/rizwanqureshi123/PDRP.

### Performance evaluation metrics

Classification model evaluation performance metrics used were precision, recall, F1-measure, and balanced accuracy defined as follows:

14 $$\begin{aligned} Precision= & {} \frac{TP}{TP + FP} \end{aligned}$$

15 $$\begin{aligned} Recall= & {} \frac{TP}{TP + FN} \end{aligned}$$

16 $$\begin{aligned} F1-measure= & {} \frac{2 \times Precision \times Recall}{Precision + Recall} \end{aligned}$$

17 $$\begin{aligned} Acc= & {} \frac{TP + TN}{TP + TN + FP + FN} \end{aligned}$$

The terms TP, FP, FN, and TN denote true positive, false positive, false negative, and true negative, respectively.

## Conclusion

Computational methods, especially machine learning^66,67^ based techniques are widely used to predict responses to lung cancer drugs. In this work, we developed a machine learning based model (PDRP), that uses clinical and demographic information (DCI), energy, and geometrical features in machine learning classifiers to predict the four levels of drug response. PDRP achieved state-of-the-art performance at 97.5% accuracy with an XGBoost classifier, even though only a small number of patients’ had information available. Our model provides a personalized drug response level prediction with a highly accurate prediction rate that could be tested on other types of cancer or other diseases. PDRP shows that modeling geometry, even in silico, can provide a powerful biomarker to predict the drug response of lung cancer patients. In future, we will further explore the dynamics and geometry of the binding-sites of protein-drug complexes. More clinical data will be collected to further refine the prediction model, and test it on other diseases.

## Supplementary Information

Supplementary Information.

## Data Availability

The datasets generated and/or analysed during the current study are not publicly available due non-disclosure agreement but are available from the corresponding author on reasonable request.
